# Willingness to adopt preventive measures among international travellers attending a vaccination centre during COVID-19: A cross-sectional study

**DOI:** 10.1016/j.aprim.2026.103516

**Published:** 2026-05-13

**Authors:** Nidia M. García-Marín, Sara Darias-Curvo

**Affiliations:** a Spanish Ministry of Health, Spain; b University of La Laguna (CEDESOG), Spain

**Keywords:** International travellers, Preventive health measures, Health communication, Health equity, Travel medicine, Viajeros internacionales, Campañas de información sanitaria, Comunicación en salud, Equidad en salud, Medicina del viajero

## Abstract

**Objective:**

To analyse international travellers’ willingness to adopt preventive health measures after the reopening of international travel following the COVID-19 pandemic, and to examine whether these intentions were aligned with expert priorities and equitably distributed across socio-demographic groups.

**Design:**

Cross-sectional observational study.

**Site:**

International Vaccination Centre of Santa Cruz de Tenerife (Spain).

**Participants:**

A total of 330 adult travellers attending pre-travel consultations between May and December 2021 (73% of all eligible travellers).

**Interventions:**

None.

**Main measurements:**

A structured questionnaire assessed travellers’ willingness to adopt preventive measures during travel. Adoption rates were compared with relevance ratings provided by a panel of 32 public health experts. Logistic regression models examined associations between preventive intentions and socio-demographic characteristics.

**Results:**

High levels of intended adoption were observed for several preventive measures, including frequent handwashing (92%; odds 12.2, 95% confidence interval [CI] 8.1–18.3), mask use in enclosed spaces (88%; odds 7.5, 95% CI 5.3–10.4), and seeking health information about the destination (78%; odds 3.5, 95% CI 2.7–4.6). The alignment between travellers’ intentions and expert priorities was strong (Pearson correlation >0.9). Multivariate logistic regression analyses showed limited socio-demographic differences in preventive intentions, with education level and occupational class not significantly associated with the willingness to adopt most preventive measures.

**Conclusions:**

Travellers reported a high willingness to adopt preventive measures after the reopening of international travel following COVID-19. Preventive intentions were strongly aligned with expert recommendations and showed minimal socio-demographic variation. These findings suggest that large-scale public health communication during the pandemic may have reached diverse traveller groups and promoted broadly equitable preventive intentions.

## Abstract (es)

**Objetivo:**

Analizar la disposición de los viajeros internacionales a adoptar medidas sanitarias preventivas tras la reapertura de los viajes internacionales en la pandemia de la COVID-19, y examinar si estas intenciones coincidían con las prioridades de los expertos y se distribuían de manera equitativa entre los distintos grupos sociodemográficos.

**Diseño:**

Estudio observacional transversal.

**Lugar:**

Centro Internacional de Vacunación de Santa Cruz de Tenerife (España).

**Participantes:**

Un total de 330 viajeros adultos que acudieron a consultas previas al viaje entre mayo y diciembre de 2021 (el 73% de todos los viajeros que cumplían los requisitos).

**Intervenciones:**

Ninguna.

**Medidas principales:**

Mediante un cuestionario estructurado se evaluó la disposición de los viajeros a adoptar medidas preventivas durante el viaje. Las tasas de adopción se compararon con las valoraciones de relevancia realizadas por un grupo de 32 expertos en salud pública. Se utilizaron modelos de regresión logística para examinar las asociaciones entre las intenciones preventivas y las características sociodemográficas.

**Resultados:**

Se observaron altos niveles de intención de adopción de varias medidas preventivas, entre ellas el lavado frecuente de manos (92%; *Odds ratio* 12,2; intervalo de confianza [IC] del 95%: 8,1-18,3), el uso de mascarilla en espacios cerrados (88%; *Odds ratio* 7,5; IC del 95%: 5,3-10,4) y la búsqueda de información sanitaria sobre el destino (78%; *Odds ratio* 3,5; IC del 95%: 2,7–4,6). La coincidencia entre las intenciones de los viajeros y las prioridades de los expertos fue elevada (correlación de Pearson > 0,9). Los análisis de regresión logística multivariante mostraron diferencias sociodemográficas limitadas en las intenciones preventivas, sin que el nivel educativo ni la categoría profesional se asociaran de forma significativa con la disposición a adoptar la mayoría de las medidas preventivas.

**Conclusiones:**

Los viajeros manifestaron una gran disposición a adoptar medidas preventivas tras la reapertura de los viajes internacionales tras la COVID-19. Las intenciones preventivas coincidían en gran medida con las recomendaciones de los expertos y mostraban una variación sociodemográfica mínima. Estos resultados sugieren que la comunicación en materia de salud pública a gran escala durante la pandemia pudo haber llegado a diversos grupos de viajeros y haber promovido intenciones preventivas ampliamente equitativas.

## Introduction

International travel to destinations with intermediate-high health risk (IHHR) exposes travellers to infectious diseases such as hepatitis A, typhoid, yellow fever, malaria, and dengue. This population represents an epidemiological priority, given the elevated risk of infection and subsequent transmission back to their countries of origin.^1^, ^2^ Many of these risks can be mitigated through individual preventive behaviors,^3^ including pre-travel vaccination and prophylaxis, as well as hygiene practices recommended by specialized travel medicine services and international vaccination centers.^4^ These services, often integrated within public health and primary care systems, play a key role in communicating preventive recommendations and supporting travellers in adopting risk-reduction behaviours.

However, decades of research show that travellers often underutilise pre-travel health advice^5^ or fail to follow it once abroad.^6^ Differences in access to information and health literacy may also create inequities in preventive behaviours among travellers.^7^ Large-scale public health information campaigns are frequently used to disseminate preventive messages to broad populations. While such campaigns aim to increase awareness and promote preventive behaviours, their influence on travellers’ intentions – particularly among international travellers – and the equity of behavioural responses remains insufficiently understood.

The COVID-19 pandemic generated an unprecedented global context of intensive public health communication. From early 2020, large-scale information campaigns promoted preventive measures such as mask wearing, hand hygiene, and social distancing.^8^, ^9^ Campaigns operated across multiple platforms – television, radio, print, digital, and social media – targeting the general population.^10^ These campaigns attempted to educate individuals on the necessity of preventive measures to reduce the probability of infection, the importance of limiting social and familial contacts, and the efficacy of vaccination against the novel coronavirus. Moreover, these communication efforts aimed to reach the entire population, thereby avoiding inequalities in knowledge and implementation of the proposed measures.^11^ Although these campaigns were not specifically directed at international travellers, they created a unique context of heightened awareness regarding infectious disease prevention.

The reopening of international travel in 2021 therefore provided an opportunity to explore travellers’ preventive intentions in this context. Rather than evaluating the effectiveness of COVID-19 communication campaigns directly, this study examines travellers’ willingness to adopt preventive measures as an indirect indicator of short-term preventive attitudes in a context of intense public health communication. Using Tenerife's International Vaccination Centre, one of the official travel medicine services within the Spanish public health system, as a case study, this study aims to:

a. assess travellers’ willingness to adopt recommended preventive measures during the early reopening of the international travel
b. examine the aligment between travellers’ willingness to adopt these measures and the priorities identified by the health experts
c. explore whether willingness to adopt preventive measures varies across socio-economic groups, potentially indicating inequities in the uptake of preventive messages.

By analysing these dimensions, the study provides insights into how travellers respond to preventive recommendations in periods of intense public health communication, with implications for travel medicine practice, primary care counselling, and the design of future health communication strategies during public health emergencies.

## Material and methods

### Study setting

The International Vaccination Centre (IVC) of Santa Cruz de Tenerife, part of Spain's Ministry of Health network, serves a population of over one million residents and handles approximately 1500 international travellers annually.

In 2021, Tenerife's IVC was staffed by three physicians and two nurses. According to data extracted from the Ministry of Health information system (SISAEX) for 2021, its size – measured in terms of the number of travellers attended and the number of medical staff – is comparable to most other Spanish IVCs, although larger centres exist in cities such as Madrid, Barcelona, and Bilbao.

Based on a set of common characteristics extracted from SISAEX (destination, travel motive, trip duration, gender, and age), the profile of travellers attending Tenerife's IVC is comparable to those attending other Spanish centres of comparable size, as documented in a previous study.^12^

### Questionnaire design and data collection

A structured anonymous questionnaire was administered to travellers attending the centre between 1 May and 31 December 2021, shortly after the reopening of international travel following the COVID-19 pandemic. The study followed a near-census approach, whereby all eligible travellers attending the International Vaccination Centre between 1 May and 31 December 2021 were invited to participate. Given this design and the finite target population, a formal a priori sample size calculation was not performed, as the objective was to include all eligible individuals attending the centre during the study period.

The questionnaire was developed through an iterative process combining previous survey instruments used in socio-economic research with items commonly collected in travel medicine practice. The development involved collaboration between the research team, physicians from the International Vaccination Centre, and researchers affiliated with the CEDESOG research centre with experience in survey design and quantitative methods.

Prior to implementation, the questionnaire underwent a two-stage pilot process. In the first phase (February 2021), the instrument was tested among health professionals and potential travellers to evaluate clarity of wording, response categories and questionnaire structure. In a second phase (March–April 2021), the questionnaire was piloted with travellers attending the centre (*n* = 15) to assess its performance under real consultation conditions. Based on feedback obtained during the pilot phases, minor revisions were introduced, including adjustments to question wording and the removal of items with low response rates.

The questionnaire was administered face-to-face at the beginning of the pre-travel consultation, prior to the delivery of travel health advice. Each interview required approximately 15 minutes to complete. Face-to-face administration facilitated identification of eligible travellers, allowed clarification of questions when necessary and contributed to high levels of response completeness.

Completed questionnaires were initially recorded on paper and subsequently transcribed into digital format by the medical staff of the centre. During this process, systematic checks were conducted to minimise transcription errors. A small number of incomplete questionnaires or cases affected by non-correctable transcription errors were excluded from the final analytical sample.

### Sample and eligibility criteria

The study targeted travellers attending the International Vaccination Centre who were planning travel to intermediate-to-high health risk (IHHR) destinations, defined as regions with documented transmission of vaccine-preventable diseases or a moderate-to-high prevalence of vector-borne diseases such as malaria or dengue.

Eligibility criteria included:

• age ≥18 years
• travel duration greater than four days
• destination in regions with endemic or high-prevalence infectious diseases
• not being airline or ship crew subject to mandatory vaccination protocols

IHHR destinations included regions of Sub-Saharan Africa, Southeast Asia, the Middle East and North Africa, and Latin America and the Caribbean.

The sampling strategy followed a near-census approach, whereby all eligible travellers attending the centre during the study period were invited to participate in the survey.

The final analytical sample consisted of 330 respondents, representing approximately 73% of all eligible travellers attending the centre during the study period (around 452 individuals).

### Expert panel

To assess the alignment between travellers’ preventive intentions and expert priorities, an expert panel was convened. The panel consisted of 32 public health professionals with experience in travel medicine and infectious disease prevention, drawn from four institutional settings – the Spanish Ministry of Health, Regional Spanish Public Health Services, The Canary Islands Primary Care Health Service (primary care physicians), and the World Health Organization – each contributing 25% of the panel members.

Experts were asked to evaluate the relevance of each preventive measure included in the traveller questionnaire using a five-point Likert scale (1 = not important, 5 = very important). The questionnaire was administered electronically between 1 May and 30 June 2022, and all invited experts completed the survey.

Mean scores for each measure were subsequently compared with the proportion of travellers reporting willingness to adopt the same preventive measures. The full questionnaire is provided in the Supplementary material.

### Variables and measurements

The questionnaire collected information across several thematic domains: socio-demographic characteristics, travel characteristics, perceived health risks, preventive behaviours, health status and socio-economic background.

Socio-demographic variables included gender, age group (<35/≥35 years), nationality, presence of children and household structure.

Socio-economic characteristics included educational attainment (primary or less, secondary, tertiary) and occupational class. Occupational information was coded using the International Standard Classification of Occupations (ISCO-08)^13^ and subsequently grouped into low, middle and high occupational categories.

Travel characteristics included destination region, travel duration and purpose of travel (e.g. tourism, work, visiting relatives or cooperation activities).

Perceived health risk variables captured travellers’ perception of the risk of contracting COVID-19 and tropical diseases during travel. Responses were grouped into low versus medium/high perceived risk categories.

Preventive behaviours were measured through questions assessing travellers’ willingness to adopt specific preventive measures during travel. These included mask use, hand hygiene practices, social distancing, accommodation choices, food and water safety, purchase of health insurance and seeking information on the health situation at the destination.

The questionnaire and the detailed coding of variables are provided in the Supplementary material.

### Statistical analysis

Descriptive statistics were used to summarise traveller characteristics and reported preventive intentions.

For each preventive measure, odds of intended adoption were calculated as the ratio between travellers reporting willingness to adopt the measure and those reporting no intention to adopt it (Yes/No). Confidence intervals for these odds were estimated based on the observed counts.

To assess the alignment between expert priorities and travellers’ intentions, scatter plots and Pearson correlation coefficients were calculated between expert scores and the percentage of travellers reporting willingness to adopt each preventive measure.

Finally, multivariate logistic regression models were estimated to analyse associations between preventive intentions and traveller characteristics, including demographic variables, socio-economic background, health status, travel characteristics and perceived health risks.

The models were estimated using maximum likelihood, and results are reported as odds ratios (OR) with corresponding confidence intervals.

## Results

### Traveller characteristics

Of 330 eligible participants (73% coverage), 175 (53%) were female and 171 (52%) were aged ≤35 years. Most travellers (208; 63%) had tertiary education, and nearly three-quarters of the sample held middle-class (112; 34%) or upper-class occupations (132; 40%). Approximately 71% reported not having children.

The primary travel purpose was tourism (204 travellers; 62%). Trip duration was relatively short, with about half of travellers (49%) planning trips of 15 days or less, while 23% planned trips longer than 30 days. Sub-Saharan Africa was the most common destination (56% of trips).

Over 83% of participants reported having received COVID-19 vaccination. There was little difference between the proportions of travellers perceiving low risk and those perceiving moderate-to-high risk, both for COVID-19 (53.9% vs 46.1%) and tropical diseases (52.7% vs 47.3%).

The full set of descriptive statistics for traveller characteristics is provided in the Supplementary online material (Table A). For more details, see also.^12^

### Adoption of preventive measures

Table 1 presents the percentages of affirmative and negative responses and the associated odds (Yes/No) for each preventive action. Odds greater than 1 indicate that the measure was more frequently intended to be adopted than not, whereas values below 1 indicate that more travellers reported not intending to adopt the measure. Statistical significance is inferred when the 95% confidence interval does not include 1.

**Table 1:** Odds of intended adoption of preventive measures among travellers.

	Yes: *n* (%)	No: *n* (%)	Odds (Yes/No)	95% conf. interval	
Wear face mask at all times	131 (40%)	199 (60%)	0.66	0.53	0.82
Wear face mask in closed spaces	291 (88%)	39 (12%)	7.46	5.34	10.42
Wear face mask in public transport	261 (79%)	69 (21%)	3.78	2.90	4.93
Wash hands frequently	305 (92%)	25 (8%)	12.20	8.11	18.34
Use disinfectant (hydroalcoholic gel)	269 (82%)	61 (18%)	4.41	3.34	5.82
More careful in maintaining social distance	251 (76%)	79 (24%)	3.18	2.47	4.09
Better and safer accommodation	184 (56%)	146 (44%)	1.26	1.01	1.57
More careful with water and food	151 (46%)	179 (54%)	0.84	0.68	1.05
Hire better health insurance	193 (58%)	137 (42%)	1.41	1.13	1.75
Information about health status	257 (78%)	73 (22%)	3.52	2.71	4.57
No change wrt previous trips	26 (8%)	304 (92%)	0.09	0.06	0.13

*Note*: Author's own calculation. Odds represent the ratio between travellers reporting intention to adopt each preventive measure and those reporting no intention (Yes/No). These odds are calculated directly from the observed frequencies and do not derive from logistic regression models. The 95% confidence intervals were computed from the observed counts. Odds greater than 1 indicate that the measure was more frequently intended to be adopted than not.

Several preventive behaviours showed very high levels of intended adoption. The most frequently reported measures were frequent hand washing (305 travellers; 92%), mask use in enclosed spaces (291 travellers; 88%), use of hydroalcoholic gel (269 travellers; 82%), maintaining social distance (251 travellers; 76%), and seeking health information about the destination (257 travellers; 78%).

In addition, 193 travellers (58%) reported intending to purchase improved travel health insurance, and 184 travellers (56%) planned to select safer accommodation options.

In contrast, some preventive actions were less commonly reported. Only 131 travellers (40%) declared an intention to wear face masks at all times, and the measure “more careful with water and food” showed no statistically significant difference between affirmative and negative responses (46% vs 54%).

Interestingly, preventive measures that received extensive attention during COVID-19 communication campaigns – such as hand hygiene, mask use in enclosed spaces, and use of disinfectant – displayed substantially higher intended adoption than traditional travel medicine precautions such as food and water safety or accommodation choices.

Finally, only 26 travellers (8%) reported no intention to modify their behaviour compared with previous trips, indicating that more than nine out of ten travellers planned to adopt at least one additional preventive behaviour following the pandemic period (Table 1).

### Alignment with expert priorities

IHHR travellers exhibited a considerable willingness to adopt preventive health measures and modify their travel practices following the reopening of international borders. A key question is whether travellers’ intended behaviours were aligned with measures considered most relevant by experts.

**Figure 1:**
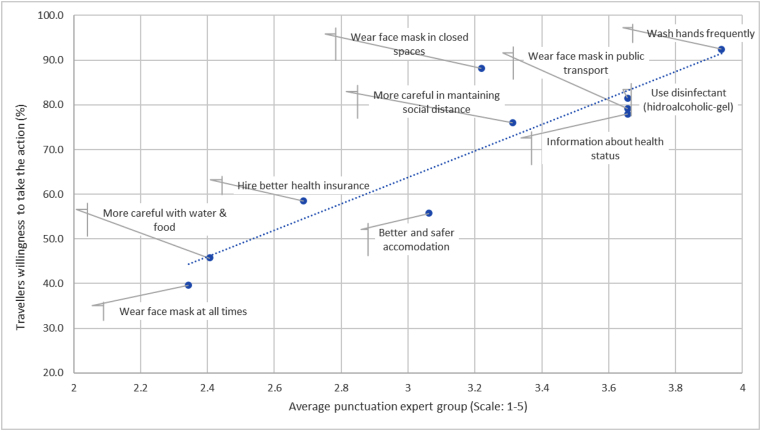
Correlation between the mean score given to each preventive measure by the group of experts and the percentage of travellers willing to adopt each measure. *Note*: Author's own calculation.

We therefore compared travellers’ responses with the priorities identified by the expert panel. Fig. 1 illustrates the relationship between expert scores and the percentage of travellers intending to adopt each measure. Experts ranked frequent hand washing, mask use on public transport, hand sanitizer use, and seeking destination health information among the most important measures.

Overall, the correspondence between expert priorities and travellers’ intentions was extremely strong, with a Pearson correlation coefficient of *r* ≈ 0.92. This indicates a high level of consistency between expert recommendations and travellers’ preventive intentions.

The measure “wearing masks in enclosed spaces” was the only one clearly above the trend line, indicating that travellers reported a higher willingness to adopt this measure than suggested by the experts’ relative prioritisation. For the remaining measures, the association between expert importance ratings and traveller adoption rates was nearly proportional.

### Health determinants and equity in the adoption of preventive measures

Multivariate logistic regression analyses explored associations between the adoption of preventive measures and traveller characteristics, controlling for travel details and perceived risk (Table 2).

**Table 2:** Logistic regression results.

		Wear face mask at all times	Wear face mask in closed spaces	Wear face mask in public transport	Wash hands frequent	Use disinfectant (hydro alcoholic-gel)	Maintain social distance	Better and safer accom.	More careful with water and food	Hire better health insuran.	Info. about health status	No change respect previous trips
**Demographic and home characteristics**												
*Nationality*	Spanish	0.848	0.248	0.133*	1.474	0.880	1.036	1.753	1.692	1.349	1.651	0.492
Omitted: non-Spanish		(0.415)	(0.275)	(0.154)	(1.503)	(0.652)	(0.512)	(0.857)	(0.736)	(0.620)	(1.013)	(0.345)
*Gender*	Male	0.898	1.014	1.055	1.093	1.207	1.118	1.020	0.785	1.350	1.102	1.241
Omitted: female		(0.246)	(0.427)	(0.339)	(0.508)	(0.366)	(0.321)	(0.280)	(0.196)	(0.360)	(0.347)	(0.639)
*Age*	≥35 years	0.902	0.514	1.282	0.725	0.699	1.129	1.098	0.977	0.574*	1.350	3.486*
Omitted (<35 years)		(0.312)	(0.319)	(0.535)	(0.620)	(0.290)	(0.439)	(0.377)	(0.299)	(0.187)	(0.524)	(2.262)
*Have children*	Yes	1.884	8.842**	2.067	1.255	3.009*	1.053	1.188	0.915	0.563	3.313*	0.582
Omitted: none		(0.840)	(7.506)	(1.382)	(1.530)	(1.840)	(0.644)	(0.563)	(0.420)	(0.258)	(2.054)	(0.450)
*Home structure*	In couple	1.506	0.745	1.671	1.052	0.924	1.391	1.241	0.844	1.405	1.099	1.127
Omitted: alone		(0.495)	(0.443)	(0.647)	(0.762)	(0.392)	(0.559)	(0.417)	(0.270)	(0.462)	(0.408)	(0.664)
	Family and child.	0.738	0.572	1.314	0.501	0.524	0.711	1.170	0.799	1.035	0.352	0.221
		(0.361)	(0.492)	(0.941)	(0.556)	(0.368)	(0.448)	(0.641)	(0.399)	(0.528)	(0.241)	(0.294)
	Other	1.080	1.253	1.380	0.292	0.765	0.836	0.916	0.813	0.625	1.437	2.146
		(0.423)	(0.782)	(0.599)	(0.243)	(0.352)	(0.343)	(0.332)	(0.299)	(0.230)	(0.592)	(1.583)

**Socio-economic characteristics**												
*Education*	Secondary	1.419	0.693	1.314	0.856	0.484	1.213	1.042	0.917	1.028	1.249	1.218
Omitted: primary		(0.724)	(0.559)	(0.715)	(0.926)	(0.357)	(0.684)	(0.553)	(0.450)	(0.440)	(0.711)	(0.820)
or less	Tertiary	1.139	0.451	1.651	0.423	0.518	0.660	0.965	0.632	1.080	2.097	1.794
		(0.597)	(0.384)	(0.947)	(0.431)	(0.381)	(0.383)	(0.515)	(0.318)	(0.481)	(1.275)	(1.271)
*Occupation class*	Mid-class	0.996	0.650	0.865	0.872	0.698	1.317	0.829	1.115	0.626	0.857	0.788
Omitted: low		(0.364)	(0.434)	(0.378)	(0.536)	(0.313)	(0.549)	(0.297)	(0.368)	(0.221)	(0.370)	(0.431)
	High-class	1.104	1.554	1.296	2.222	0.905	1.160	1.213	0.618	0.738	0.656	0.454
		(0.435)	(1.027)	(0.643)	(1.545)	(0.437)	(0.510)	(0.492)	(0.232)	(0.288)	(0.328)	(0.289)

**Individual health aspects**												
*Health status*	Good–very good	0.964	2.190	1.999	2.033	0.994	1.514	1.186	1.696	0.927	1.150	1.856
Omitted: mid-low		(0.406)	(1.654)	(0.937)	(1.661)	(0.587)	(0.713)	(0.569)	(0.699)	(0.373)	(0.646)	(1.795)
*Had disease*	Yes	2.109**	1.350	2.251	0.800	1.322	1.568	2.196**	0.890	2.305**	1.402	1.633
Omitted: no		(0.758)	(1.085)	(1.342)	(0.641)	(0.709)	(0.691)	(0.837)	(0.313)	(0.922)	(0.699)	(1.239)
*Vaccine COVID*	Yes	3.575***	10.59***	1.889*	2.985*	2.589**	1.785	4.283***	1.625	1.228	0.668	1.112
Omitted: no		(1.532)	(5.765)	(0.707)	(1.790)	(1.083)	(0.642)	(1.543)	(0.601)	(0.476)	(0.306)	(0.684)

**Travel characteristics**												
*Destiny*	Rest Asia and N. Afr.	0.789	0.114**	0.782	0.286	0.335	0.189	0.131*	0.534	0.189**	1.121	3.428
Omitted: C. Amer.		(0.704)	(0.110)	(0.818)	(0.376)	(0.348)	(0.210)	(0.145)	(0.512)	(0.148)	(1.371)	(4.294)
	S. America	0.742	0.132***	1.167	0.826	0.519	0.707	0.488*	0.467*	0.697	1.374	2.301
		(0.311)	(0.0921)	(0.528)	(0.631)	(0.251)	(0.325)	(0.210)	(0.184)	(0.303)	(0.720)	(1.924)
	S. East Asia	0.182**	0.325	0.933	1.378	0.912	0.537	0.242**	1.130	1.308	1.307	–
		(0.136)	(0.352)	(0.718)	(1.581)	(0.690)	(0.344)	(0.169)	(0.706)	(0.929)	(1.119)	–
	SSA	1.470	0.791	2.073*	1.196	1.051	0.891	0.391**	0.531*	0.594	0.368**	2.329
		(0.548)	(0.488)	(0.873)	(0.865)	(0.462)	(0.365)	(0.156)	(0.189)	(0.218)	(0.167)	(1.663)
*Motive*	Tourist	0.484**	0.942	1.532	0.485	0.702	1.471	0.582	0.393**	2.334**	0.510	0.909
Omitted: other		(0.177)	(0.535)	(0.623)	(0.413)	(0.305)	(0.580)	(0.215)	(0.147)	(0.869)	(0.228)	(0.476)
	Work	1.190	0.829	1.332	3.038	1.995	1.666	1.068	0.755	1.789	1.538	0.444
		(0.503)	(0.543)	(0.694)	(2.598)	(1.200)	(0.755)	(0.480)	(0.316)	(0.785)	(0.820)	(0.329)
*Duration*	16–30 days	1.089	1.079	0.809	0.951	0.983	1.057	0.750	0.975	0.567*	0.253***	3.783**
Omitted: ≤15 days		(0.345)	(0.527)	(0.275)	(0.502)	(0.356)	(0.336)	(0.231)	(0.297)	(0.169)	(0.0921)	(2.207)
	>30 days	1.894	0.577	1.191	0.442	0.482	0.844	0.578	0.800	0.720	0.300**	3.460
		(0.816)	(0.395)	(0.596)	(0.376)	(0.272)	(0.399)	(0.259)	(0.325)	(0.307)	(0.161)	(2.661)

**Risk perception during the travel**												
*Risk percep. COVID*	Mid-high	1.577	4.618***	1.056	1.294	1.756	1.163	2.498***	1.479	1.509	1.006	0.604
Omitted: low		(0.465)	(2.195)	(0.334)	(0.754)	(0.634)	(0.335)	(0.713)	(0.384)	(0.407)	(0.333)	(0.287)
*Risk percep.* *Tropic*	Mid-high	1.007	1.667	1.246	2.358	1.712	1.369	1.195	1.403	1.352	0.993	0.584
Omitted: low		(0.304)	(0.756)	(0.398)	(1.317)	(0.588)	(0.399)	(0.348)	(0.362)	(0.372)	(0.322)	(0.320)
*Pseudo-R*^*2*^		0.131	0.264	0.098	0.161	0.102	0.054	0.128	0.072	0.107	0.103	0.150
*Num. obs.*		330	330	330	330	330	330	330	330	330	330	312

*Notes*: Author's own calculation. The table reports the association between individual, socio-demographic, health status and travel characteristics with travellers’ willingness to adopt each preventive measure. Coefficients correspond to the estimated effects from the logistic regression models described in the Methods section. Pseudo-R^2^ indicates the proportion of variation in the outcome explained by the model, using the standard goodness-of-fit measure for logistic regression models. Standard errors are shown in parentheses. *Abbreviations*: OR, odds ratio; CI, confidence interval. Statistical significance is indicated as follows.

* *p* < 0.05.

** *p* < 0.01.

*** *p* < 0.001.

Drawing on the literature on health inequalities^14^, ^15^, ^16^, ^17^ and equality of opportunity,^18^, ^19^, ^20^ demographic and socio-economic characteristics are particularly relevant because they are often associated with structural inequalities beyond individual control.

Results showed that socio-economic variables such as education level and occupational class were not statistically significant predictors for the majority of preventive behaviours. Across the multivariate models, only a small proportion of estimated coefficients were statistically different from 1, indicating limited socio-demographic variation in preventive intentions.

A limited number of other associations reached statistical significance:

• Travellers with children had substantially higher odds of intending to wear masks in enclosed spaces (OR ≈ 8.8), use hydroalcoholic gel (OR ≈ 3.0), and seek destination health information (OR ≈ 3.3).
• Travellers aged ≥35 years were less likely to plan purchasing enhanced travel health insurance compared with younger travellers.
• Nationality was generally not associated with preventive intentions, although Spanish nationals were less likely to report mask use on public transport.
• Travellers vaccinated against COVID-19 showed significantly higher odds of adopting several preventive behaviours, including mask use in enclosed spaces (OR > 10) and selecting safer accommodation options (OR ≈ 4.3).
• Travel destination showed few systematic differences, although some regional variations were observed for accommodation choices and mask use.
• When the purpose of travel was tourism, travellers were more likely to report purchasing better health insurance but less likely to wear face masks at all times or report greater caution regarding food and water safety.
• Higher perceived risk of COVID-19 during travel was associated with higher odds of mask use in enclosed spaces (OR ≈ 4.6) and other preventive behaviours.

Overall, the limited number of statistically significant socio-demographic associations reinforces that preventive intentions were broadly consistent across traveller groups, suggesting relatively equitable uptake of preventive health messages within the study population.

## Discussion

This study examined international high-health-risk (IHHR) travellers’ willingness to adopt preventive health measures during the reopening of international travel after the COVID-19 pandemic. Overall, travellers reported a high willingness to adopt recommended preventive behaviours, particularly frequent handwashing, mask use in enclosed spaces, and seeking health information about their destination. These patterns were consistent with the priorities identified by the panel of public health experts, indicating a substantial alignment between travellers’ preventive intentions and expert recommendations.

Interestingly, measures that received intensive visibility during COVID-19 communication campaigns, such as hand hygiene and mask use, showed substantially higher intended adoption than more traditional travel medicine precautions such as food and water safety. This pattern is consistent with the influence of sustained public health communication on travellers’ preventive attitudes.

Although the study does not allow causal inference regarding the effectiveness of specific public health campaigns, the patterns observed are consistent with the possibility that large-scale communication efforts during the pandemic may have contributed to shaping preventive intentions in this population. Unlike many public health interventions that struggle to engage disadvantaged groups and thus exacerbate inequalities, our findings suggest that, within the study population, preventive messages during the COVID-19 pandemic reached travellers broadly across socio-demographic profiles. The absence of strong socio-economic gradients in willingness to adopt preventive measures is noteworthy. Consistent with this pattern, the multivariate analyses showed that education level and occupational class were not significant predictors for most preventive behaviours. Preventive health behaviours often follow social gradients in the general population.^21^, ^22^ Taken together, these findings suggest that intensive public health communication may help reduce disparities in access to preventive information and behavioural uptake, as highlighted in previous research on health communication strategies.^23^

These findings should also be interpreted in relation to previous research on preventive behaviours during the COVID-19 pandemic. Several studies conducted in the general population reported relatively high adherence to preventive measures during the early phases of the pandemic, particularly among individuals perceiving higher levels of health risk.^24^, ^25^ However, evidence focusing specifically on international travellers during the reopening of global mobility remains limited. In this sense, the present study provides a snapshot of preventive intentions among travellers immediately after the lifting of travel restrictions, a period characterised by intense public health communication and heightened awareness of infectious disease risks.

Future research should explore the durability and generalizability of these effects. Longitudinal studies are needed to assess whether preventive behaviors persist after travel and beyond acute crises, such as the declaration of the end of the public health emergency of international concern in May 2023. Comparative analyses across different traveller groups and campaign formats would also clarify which communication strategies are most effective in achieving sustained impact.

The broader implications extend beyond the COVID-19 context. Public health systems can integrate preventive communication strategies targeting travellers into routine travel medicine and emergency preparedness frameworks. In many regions where specialised vaccination centres are limited, travel health counselling is frequently delivered within primary care services. In these contexts, primary care professionals may play an important role in reinforcing preventive messages and supporting the adoption of protective behaviours before travel. Structured travel-health information protocols coordinated with nearby International Vaccination Centres could help standardise preventive advice, reinforce public health messages across clinical settings, and ensure that preventive information reaches travellers consistently across socio-demographic groups. Pre-established mechanisms for rapid deployment, multi-channel delivery, and continuous monitoring are essential to ensure both effectiveness and fairness in protection.

Several limitations should be acknowledged. First, the study population was limited to IHHR travellers attending a single international vaccination center, a subgroup that may not fully represent the general traveling population. These travellers could exhibit a greater predisposition toward risk awareness, limiting the generalizability of the findings. Nonetheless, the high response rate and the fact that the centre serves a large and diverse population of travellers within the Spanish public health system support the internal validity of the results.

Second, outcomes were based on self-reported intentions rather than observed behaviors, which may not always translate into action. Behavioural science research has long documented a gap between intentions and behaviour; however, behavioural intention remains one of the strongest predictors of subsequent action and is commonly used as an indicator of behavioural adoption in preventive health research.^26^, ^27^ In addition, potential sources of bias should be considered when interpreting the results. The study population consisted of travellers who proactively sought pre-travel consultation and may therefore be more health-conscious than the general travelling population, which could introduce selection bias. Moreover, the use of self-reported intentions may be affected by social desirability bias, as respondents may overstate their willingness to adopt recommended preventive behaviours. Taken together, these factors suggest that the reported levels of preventive intentions may represent an upper-bound estimate of actual behaviour.

Third, the survey was conducted during the early phase of international travel reopening after the COVID-19 pandemic, and findings cannot be directly extrapolated to present-day practices. Indeed, preventive intentions among travellers may have evolved since the period of intense public health communication observed during the pandemic. As risk perception declines and media attention shifts, adherence to preventive behaviours may also change, potentially leading to lower willingness to adopt certain preventive measures and the re-emergence of socio-demographic differences in access to preventive information. This possibility reinforces the importance of maintaining structured and evidence-based health communication strategies in travel medicine settings to sustain preventive awareness over time.

## Conclusion

This study shows that international travellers attending a vaccination centre during the reopening of international travel after COVID-19 reported a high willingness to adopt recommended preventive measures, with intentions closely aligned with expert priorities and showing limited socio-demographic variation.

Although causal conclusions about the effectiveness of specific public health campaigns cannot be drawn from this observational study, the patterns observed are consistent with the possibility that large-scale health communication efforts during the pandemic may have contributed to shaping preventive intentions in this population.^28^ This is an encouraging outcome given the diversity, mobility, and potential vulnerability of such traveller groups.

The originality of this study lies in showing that preventive intentions reported by travellers were strongly aligned with expert recommendations and broadly consistent across socio-demographic groups. The absence of strong socio-economic gradients in these intentions is noteworthy, given that many public health interventions tend to display unequal uptake across social groups. These results suggest that large-scale health communication strategies may have the potential to reach diverse population groups relatively evenly.^29^

The practical implications extend beyond the pandemic. Public health communication strategies aimed at travellers may play an important role in promoting preventive awareness before travel, particularly when integrated into routine travel health consultations, vaccination centres, pharmacies, and other settings involved in travel medicine. In settings where specialised travel medicine services are limited, primary care professionals may play a key role in delivering and reinforcing these preventive messages. Ensuring that communication strategies remain accessible and inclusive across socio-economic, cultural and linguistic groups is essential to avoid reinforcing existing health inequalities.

From a policy perspective, these findings highlight the importance of maintaining institutional capacity to deliver clear, coordinated and multi-channel public health messages during future health emergencies. Establishing frameworks for rapid deployment of evidence-based communication strategies, supported by monitoring systems and feedback mechanisms, may help public health authorities respond more effectively and equitably.

Finally, future research should continue to evaluate health communication strategies not only in terms of reach and stated adoption but also in relation to actual behavioural outcomes and their impact on health inequalities. By integrating equity-focused communication into both emergency preparedness and routine travel medicine, health systems can better support preventive behaviours among mobile populations and contribute to protecting global public health.

Strengthening equitable and well-coordinated health communication strategies in travel medicine and primary care settings can play a key role in promoting preventive behaviours among travellers and improving preparedness for future global health threats.

**What is known about the topic?**

International travellers to high-risk destinations often underuse or ignore preventive advice. Reviews show that large-scale health information campaigns can improve awareness, but their reach is uneven, and little is known about their equity effects in travel medicine.

**What does the study add to the literature?**

This study demonstrates that a large-scale campaign promoted high adoption of expert-recommended preventive measures among international travellers, with minimal socio-demographic disparities.

**What are the implications of the results obtained?**

Health information campaigns, when clear and inclusive, can be integrated into routine travel medicine to ensure equitable uptake, strengthen preparedness, and reduce inequalities in future health crises.

## Authors’ contributions

Nidia M. García-Marín: study conception, data collection, data interpretation, drafting of manuscript, critical review of manuscript. Sara Darias-Curvo: study conception, supervision, data interpretation, drafting of manuscript, critical review of manuscript. All authors approved the final version.

## Ethical approval

Approval was obtained from the Research Ethics and Animal Welfare Committee of University of La Laguna (CEIBA 2023-3357). http://sede.ull.es/validacion; Código de verificación: C/tM85K2.

All participants provided informed consent.

## Declaration of generative AI and AI-assisted technologies in the writing process

The authors declare that no artificial intelligence tools were used in the design of the study, data collection, data analysis, or manuscript writing.

## Funding

This research received no specific grant from any funding agency, public, commercial, or not-for-profit sectors.

## Conflict of interest

The authors declare no conflicts of interest.

## References

[1] Angelo K.M., Kozarsky P.E., Ryan E.T., Chen L.H., Sotir M.J. (2017). What proportion of international travellers acquire a travel-related illness? A review of the literature. J Travel Med.

[2] Freedman D., Weld L., Kozarsky P., Fisk T., Robins R., Sonnenburg F. (2006). Spectrum of disease and relation to place of exposure among ill returned travelers. N Engl J Med.

[3] Farnham A., Baroutsou V., Hatz C., Fehr J., Kuenzli E., Blanke U. (2022). Travel behaviours and health outcomes during travel: profiling destination-specific risks in a prospective mHealth cohort of Swiss travellers. Travel Med Infect Dis.

[4] Hill D.R., Ericsson C.D., Pearson R.D., Keystone J.S., Freedman D.O., Kozarsky P.E. (2006). The practice of travel medicine: guidelines by the Infectious Diseases Society of America. Clin Infect Dis.

[5] LaRocque R.C., Rao S.R., Tsibris A., Lawton T., Barry M.A., Marano N. (2010). Pre-travel health advice-seeking behavior among US international travelers departing from Boston Logan International Airport. J Travel Med.

[6] Lammert S., Rao S., Jentes E., Fairley J., Erskine S., Walker A. (2016). Refusal of recommended travel-related vaccines among U.S. international travellers in Global TravEpiNet. J Travel Med.

[7] Schlagenhauf P., Weld L., Goorhuis A., Gautret P., Weber R., von Sonnenburg F. (2015). Travel-associated infection presenting in Europe (2008–2012): an analysis of EuroTravNet longitudinal surveillance data. Lancet Infect Dis.

[8] WHO. Advice for the public: Coronavirus disease (COVID-19). Available from: https://www.who.int/emergencies/diseases/novel-coronavirus-2019/advice-for-public.

[9] Ministerio de Sanidad España (2021). https://www.sanidad.gob.es/areas/alertasEmergenciasSanitarias/alertasActuales/nCov/ciudadania.htm.

[10] Moreno A., Fuentes C., Navarro C. (2020). Covid-19 communication management in Spain: exploring the effect of information-seeking behavior and message reception in public's evaluation. El Profesional de la Información.

[11] Kalocsányiová E., Essex R., Fortune V. (2023). Inequalities in Covid-19 messaging: a systematic scoping review. Health Commun.

[12] García-Marín N.M., Marrero G.A., Guerra-Neira A., Rivera-Deán A. (2023). Profiles of travelers to intermediate-high health risk areas following the reopening of borders in the COVID-19 crisis: a clustering approach. Travel Med Infect Dis.

[13] International Labour Office (2012).

[14] CSDH (2008).

[15] Dahlgren G., Whitehead M. (2006).

[16] Marmot M. (2013).

[17] Whitehead M., Dahlgren G. (2006).

[18] Bricard D., Jusot F., Trannoy A., Tubeuf S. (2013). Inequality of opportunities in health and the principle of natural reward: evidence from European countries. Res Econ Inequality.

[19] Roemer J. (2002). Equality of opportunity: a progress report. Soc Choice Welfare.

[20] Marrero G.A., Rodríguez J.G. (2012). Inequality of opportunity in Europe. Rev Income Wealth.

[21] Marmot M. (2015).

[22] Bambra C., Riordan R., Ford J., Matthews F. (2020). The COVID-19 pandemic and health inequalities. J Epidemiol Community Health.

[23] Michie S., van Stralen M.M., West R. (2011). The behaviour change wheel: a new method for characterising and designing behaviour change interventions. Implement Sci.

[24] Dryhurst S., Schneider C.R., Kerr J., Freeman A.L.J., Recchia G., van der Bles A.M. (2020). Risk perceptions of COVID-19 around the world. J Risk Res.

[25] Clark C., Davila A., Regis M., Kraus S. (2020). Predictors of COVID-19 voluntary compliance behaviors: an international investigation. Global Transit.

[26] Ajzen I. (1991). The theory of planned behavior. Org Behav Human Decis Process.

[27] Sheeran P. (2002). Intention–behavior relations: a conceptual and empirical review. Eur Rev Soc Psychol.

[28] Kreps G.L., Maibach E.W. (2008). Transdisciplinary science: the nexus between communication and public health. J Commun.

[29] Wakefield M.A., Loken B., Hornik R.C. (2010). Use of mass media campaigns to change health behaviour. Lancet.

