# Supplementary material for: Willingness to adopt preventive measures among international travellers attending a vaccination centre during COVID-19: A cross-sectional study
Source: Aten Primaria. 2026 May 13;58(8):103516. doi: 10.1016/j.aprim.2026.103516 (PMC13196378; doi:10.1016/j.aprim.2026.103516)
Supplement: Supplementary file 1 [file mmc1.doc]

Supplementary material

Table A: Travellers average characteristics (%)

| Variable | Obs (n) | Mean (%) | Std. Dev. |
| --- | --- | --- | --- |
|  |  |  |  |
| Nationality |  |  |  |
| Non-Spanish | 330 | 8,78 | 28,35 |
| Spanish | 330 | 91,21 | 28,35 |
| Gender |  |  |  |
| Female | 330 | 52,72 | 50 |
| Male | 330 | 47,27 | 50 |
| Age |  |  |  |
| < 35 | 330 | 52,42 | 50,01 |
| ≥ 35 | 330 | 47,57 | 50,01 |
| Children |  |  |  |
| No | 330 | 73,93 | 43,96 |
| Yes | 330 | 26,06 | 43,96 |
| Home Structure |  |  |  |
| Alone | 330 | 29,69 | 45,76 |
| In couple | 330 | 31,21 | 46,4 |
| Family-children | 330 | 16,66 | 37,32 |
| Other | 330 | 22,42 | 41,77 |
| Education |  |  |  |
| Primary or less | 330 | 9,69 | 29,63 |
| Secondary | 330 | 27,57 | 44,75 |
| Tertiary | 330 | 62,72 | 48,42 |
| Occupation class |  |  |  |
| Low | 330 | 25,15 | 43,45 |
| Mid-class | 330 | 34,54 | 47,62 |
| High-class | 330 | 40,3 | 49,12 |
| Health status |  |  |  |
| Mid-low | 330 | 9,69 | 29,63 |
| Good-very good | 330 | 90,3 | 29,69 |
| Had disease |  |  |  |
| No | 330 | 84,24 | 36,46 |
| Yes | 330 | 15,75 | 36,48 |
| Vaccine COVID-19 |  |  |  |
| No | 330 | 16,06 | 36,77 |
| Yes | 330 | 83,93 | 36,77 |
| Destiny |  |  |  |
| Central America | 330 | 16,66 | 37,32 |
| Rest Asia & North Africa | 330 | 1,81 | 13,38 |
| South America | 330 | 20 | 40,06 |
| South East Asia | 330 | 5,45 | 22,74 |
| Subsaharian Africa (SSA) | 330 | 56,06 | 49,7 |
| Motive |  |  |  |
| Tourist | 330 | 62,42 | 48,5 |
| Work | 330 | 19,39 | 39,59 |
| Other | 330 | 18,18 | 38,62 |
| Duration |  |  |  |
| ≤ 15 days | 330 | 51,21 | 50,06 |
| 16-30 days | 330 | 30,9 | 46,28 |
| > 30 days | 330 | 17,87 | 38,37 |
| Risk perception COVID-19 |  |  |  |
| Low | 330 | 53,93 | 49,92 |
| Mid-high | 330 | 46,06 | 49,92 |
| Risk perception tropical |  |  |  |
| Low | 330 | 52,72 | 50 |
| Mid-high | 330 | 47,27 | 50 |
